# Targeting prosurvival BCL2 signaling through Akt blockade sensitizes castration‐resistant prostate cancer cells to enzalutamide

**DOI:** 10.1002/pros.23843

**Published:** 2019-06-22

**Authors:** Amanda B. Pilling, Clara Hwang

**Affiliations:** ^1^ Department of Internal Medicine, Division of Hematology/Oncology, Henry Ford Health System Henry Ford Cancer Institute Detroit Michigan

**Keywords:** apoptosis, androgen receptor antagonist, castration‐resistant prostate cancer, PI3K

## Abstract

**Background:**

Prostate cancer that recurs after initial treatment inevitably progresses to castration‐resistant prostate cancer (CRPC), the lethal stage of the disease. Despite improvements in outcomes from next generation androgen receptor (AR)‐axis inhibitors, CRPC remains incurable. Therapeutic strategies to target AR antagonist resistance are urgently needed to improve outcomes for men with this lethal form of prostate cancer.

**Methods:**

Apoptosis and BCL2 family signaling were characterized in cell line models of CRPC. Quantitative real‐time polymerase chain reaction and Western blot analysis were used to determine BCL2 expression levels. Drug sensitivity was determined by proliferation, survival and apoptosis analysis. Protein‐protein interactions were evaluated by coimmunoprecipitation followed by Western blot detection.

**Results:**

In the present study, we identify antiapoptotic BCL2 protein signaling as a mechanism of resistance to AR antagonist enzalutamide. In CRPC cell line models, we found that BCL‐xL and MCL‐1 proteins block apoptosis through binding and sequestering proapoptotic proteins BIM and BAX, resulting in cell survival in response to enzalutamide. Treatment with BH3‐mimetics targeting BCL‐xL or MCL‐1 disrupts these interactions and activates apoptosis, sensitizing CRPC cells to enzalutamide. Importantly, we demonstrate that PI3K/Akt signaling is activated in response to enzalutamide and mediates apoptosis evasion through inactivation of BAD, a BH3‐only protein that activates proapoptotic signlaing through inhbition of BCL‐xL. Inhibition of Akt activates BAD, resulting in increased apoptosis and sensitivity to enzalutamide, demonstrating an alternative therapeutic strategy to target drug resistance.

**Conclusions:**

These results demonstrate that CRPC cells employ multiple mechanisms to mediate apoptosis evasion through BCL2 signaling, suggesting this pathway is critical for survival. This study provides a strong preclinical rationale for developing therapeutic strategies to target antiapoptotic BCL2 signaling in combination with AR antagonists to improve treatment options for patients with advanced prostate cancer.

## 1 INTRODUCTION

Prostate cancer is the most commonly diagnosed cancer and the second leading cause of cancer death in American men. Despite high response rates to initial androgen deprivation therapy, most patients with advanced disease will progress to metastatic castration‐resistant prostate cancer (mCRPC). First‐line treatments for mCRPC include targeting the androgen receptor (AR) axis with next‐generation AR signaling inhibitors such as abiraterone acetate and enzalutamide (ENZ). While these therapies provide clinically meaningful benefits, responses are not durable and patients eventually progress on treatment.^1^, ^2^ Many patients exhibit primary (de novo) drug resistance, and patients that do respond will inevitably develop resistance, leading to disease progression and limited survival. Therefore, it is critical to identify improved therapeutic strategies for this lethal form of prostate cancer.

Activation of apoptosis in response to anticancer therapies is known to be a critical mechanism to reduce tumor burden and achieve favorable therapeutic response rates. For the treatment of prostate cancer, several early studies demonstrated that the initial rapid responses and tumor involution observed in response to androgen withdrawal is mediated by apoptotic cell death.^3^ Moreover, it was shown that AR inhibition with ENZ induces tumor regression through apoptosis in CRPC xenograft studies.^4^ Our group recently demonstrated that targeting inhibitor of apoptosis proteins (cIAP1/2), which block death receptor (or extrinsic) pathway signaling, increases response and sensitivity to ENZ in CRPC cell line models, providing further evidence that engaging apoptosis is likely critical to increasing activity of AR antagonists.^5^

The intrinsic (or mitochondrial) pathway of apoptosis is a major apoptotic mechanism regulated by the BCL2 family proteins through a complex network of protein‐protein interactions. The BCL‐2 family consists of antiapoptotic BCL‐2, BCL‐xL, MCL‐1, BCL‐w, proapoptotic effectors BAX and BAK, and the proapoptotic BH3‐only proteins BIM, BID, BAD, PUMA, and NOXA. The balance of the proapoptotic and antiapoptotic family members determines whether BAX and BAK are activated leading to mitochondrial outer membrane permeabilization (MOMP), the release of cytochrome *c* and second mitochondria‐derived activator of apoptosis, followed by caspase‐9 activation, culminating in cell‐wide proteolysis and death.^6^

Antiapoptotic BCL‐2 proteins are frequently overexpressed in cancer and are associated with an aggressive, treatment‐refractory disease. In prostate cancer, several studies demonstrate that overexpression of antiapoptotic BCL2 proteins are adverse prognostic factors associated with disease progression and therapy resistance.^7^, ^8^, ^9^ Increased expression of these antiapoptotic proteins can suppress apoptosis by sequestering the proapoptosis players and preventing activation BAX and BAK. Therefore, targeting the antiapoptotic BCL‐2 proteins is an attractive strategy to lower the apoptotic threshold and increase therapeutic response in prostate tumors. In this study, we identify the BCL2 family proteins that block apoptosis in response to ENZ and identify multiple strategies to target these proteins and enhance the action of ENZ in CRPC cell line models.

## 2 MATERIALS AND METHODS

### 2.1 Cell lines and reagents

LNCaP and 22Rv1 cells were obtained from the American Type Culture Collection in 2012 (ATCC). C4‐2 cells were obtained from MD Anderson Cancer Center Cell Line Core Facility in 2016 (Houston, TX). All cells were maintained in Rosewell Park Memorial Institute supplemented with 10% fetal bovine serum. Cell line authentication was performed using short tandem repeat profiling (GenePrint 10 kit, Promega). Mycoplasma detection is performed on a plate luminometer using a mycoplasma enzyme‐based luciferase assay (MycoAlert PLUS Mycoplasma Detection Kit, Lonza). Low‐passage (<15) cultures were used for all experimental testing, Enzalutamide (MDV3100), venetoclax (ABT‐199), navitoclax (ABT‐263), A‐1210477, obatoclax, MK2206, and buparlisib were purchased from Selleck Chemicals. Antibodies for Western blot analysis include glyceraldehyde 3‐phosphate dehydrogenase (GAPDH) (sc‐365062) and tubulin (sc‐8035): Santa Cruz Biotechnologies; NOXA (114C307): Novus Biologicals; PARP‐1 cleaved (5625), BCL‐2 (4223), BCL‐xL (2764), MCL‐1 (5433), BAX (5023), BIM 2933), BAD (9239), pBAD‐Ser136 (4366), Akt (4691), and pAkt‐Ser473 (4060): Cell Signaling Technology.

### 2.2 Viability assays

Viability was measured using the CellTiter‐GLO luminescent assay according to the manufacturer's instructions (Promega). Briefly, cells were seeded into 96‐well plates at a density to permit exponential growth throughout the length of the assay 24 hours before drug treatment. Viability was detected by luminescent signal 72 hours after drug treatment using a Victor X1 Luminescence Plate Reader (Perkin Elmer). Viability is displayed as percent of the untreated control. IC_50_ values were calculated using Prism v5.02 (GraphPad, San Diego, CA).

### 2.3 Clonogenic survival

Cells were seeded into six‐well plates at a density to permit exponential growth throughout the length of the assay 24 hours before drug treatment. Cells were treated every 72 hours over the course of 14 days after which surviving colonies were stained with 0.1% crystal violet and quantified using ImageJ software.

### 2.4 Western blot analysis

Immunoblotting was conducted as previously described with minor modifications.^10^ Briefly, cells were lysed in radioimmunoprecipitation assay (RIPA) buffer supplemented with Halt Protease and Phosphatase Inhibitor Cocktail. Total protein was separated by sodium dodecyl sulfate‐polyacrylamide gel electrophoresis, transferred to nitrocellulose, and stained with the indicated primary antibodies followed by horseradish peroxidase‐linked secondary antibodies. Protein visualization was achieved by enhanced chemiluminescence detection.

### 2.5 Protein complex immunoprecipitation

Following the indicated drug treatments, cells were lysed with modified RIPA buffer (50 mM Tris‐HCl, 1% NP‐40, 0.25% Na deoxycholate, 150 mM NaCl, 1 mM ethylenediaminetetraacetic acid). Protein (250 µg) was incubated overnight with rotation with 2.5 µg of indicated antibodies (Cell Signaling Technology): BIM (2933), BCL‐xL (2764), or MCL‐1 (94296). Immunocomplexes were recovered with 20 µL protein G agarose beads (Cell Signaling Technology). Protein complexes were separated and solubilized by boiling in 2× Laemmli buffer.

### 2.6 Flow cytometry

Analysis of apoptosis by flow cytometry was measured after cells were treated for 72 hours. Cells were washed and double stained with annexin V‐fluorescein isothiocyanate and propidium iodide (BD Biosciences) followed by flow cytometry analysis. The mitochondrial transmembrane potential (ΔΨ_m_) was determined using JC‐1 Mitoscreen (BD Biosciences). Cells were harvested after the indicated treatment and stained with JC‐1, washed, and subjected to detection by flow cytometry. Detection of JC‐1 aggregates was dependent on JC‐1 concentration, where higher concentrations indicated accumulation of JC‐1 aggregates within polarized (intact) mitochondria membrane. All flow cytometric analysis were performed using a BD LSR II flow cytometer and data were analyzed using FlowJo software (Becton, Dickinson and Company).

### 2.7 Quantitative real‐time polymerase chain reaction

Total RNA (1ug) was reverse‐transcribed using High Capacity cDNA Reverse Transcription Kit from Life Technologies. Complementary DNA (1 μl) was PCR amplified in a 20 μl reaction including TaqMan 2X Universal Master Mix and TaqMan gene expression probe/primer set for *BCL‐2, BCL‐xL, MCL‐1, BAX, BIM, NOXA*, and *GAPDH*. The quantitative polymerase chain reaction reactions were run using ABI‐7500 FAST real‐time PCR system (Applied Biosystems). Samples were run in triplicate for a total of three separate experiments.

### 2.8 Statistical analysis

Statistical significance was assessed by the Student *t* test (two‐tailed distribution, two‐sample, and unequal variance) and considered statistically significant with *P* < .05.

## 3 RESULTS

### 3.1 Mitochondrial apoptosis is activated in response to AR antagonist

**Figure 1:**
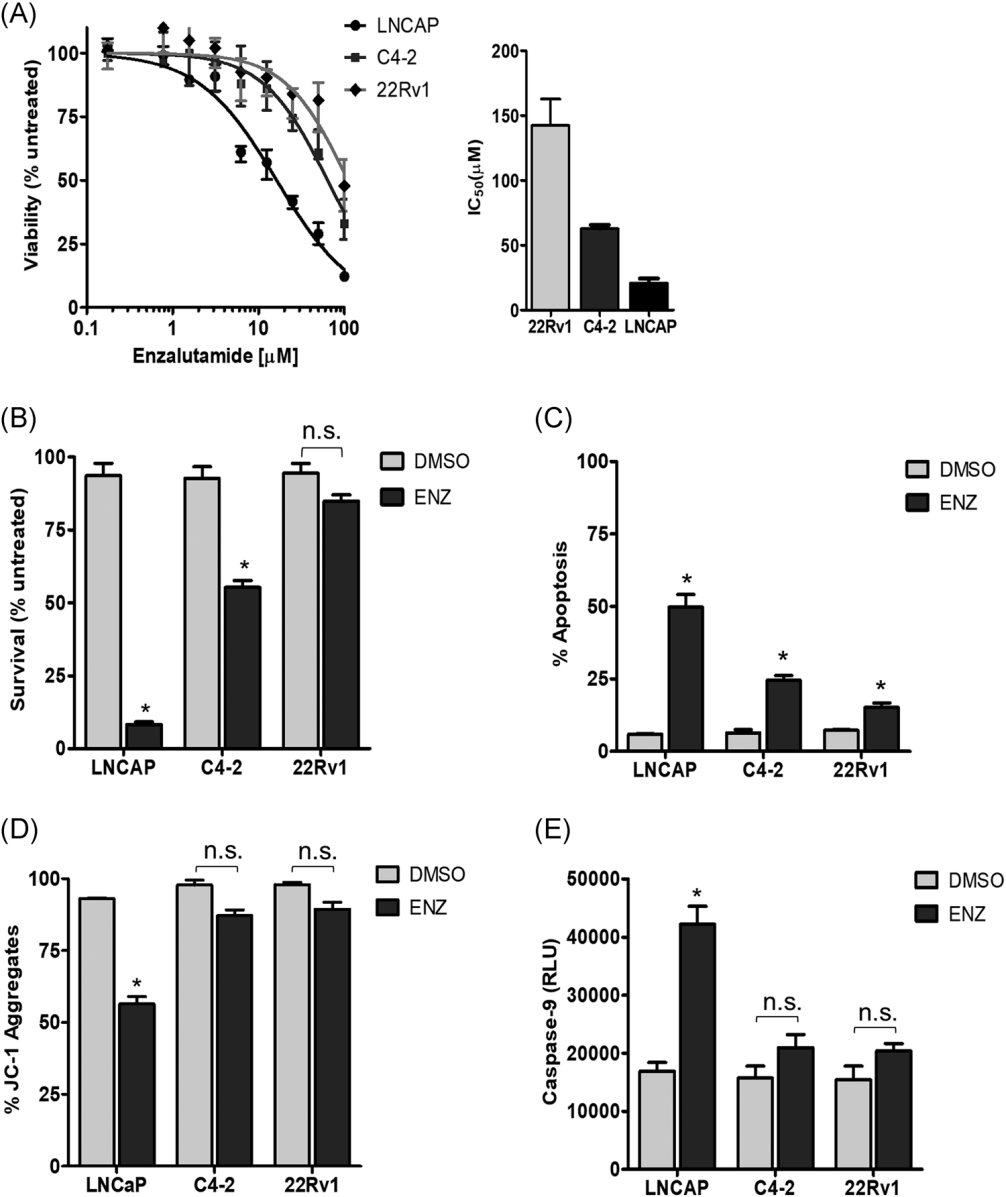
Mitochondrial apoptosis is activated in response to enzalutamide in AR‐dependent cells. A, Cell viability was measured in response to ENZ or vehicle control, shown as percent of vehicle‐treated control. IC_50_ values are shown for the indicated cell line. B, Clonogenic survival of cell lines at 14 days of treatment with 20 µM ENZ compared to vehicle‐treated control. C, Apoptosis measured by annexin‐V staining in cells treated with 20 µM ENZ or vehicle quantified by flow cytometry. D, Mitochondrial membrane potential (ΔΨ_m_) in cells treated with 20 µM ENZ or vehicle control. Percent of JC‐1 aggregates determined by flow cytometry is shown. E, Caspase‐9 activity in cells treated with 20 µM ENZ or vehicle control. Values represent the mean ± SEM from three independent experiments. AR, androgen receptor; DMSO, dimethyl sulfoxide; ENZ, enzalutamide. *P < .05

Several early studies have demonstrated that apoptotic cell death is critical for response to androgen axis inhibition.^3^, ^4^ To further establish the link between apoptosis and sensitivity to AR antagonism, we measured viability, survival, and apoptosis in response to ENZ in a panel of prostate cancer cell lines. The cell lines tested included androgen‐dependent LNCAP cells and CRPC cell lines C4‐2 and 22Rv1. We first determined viability and the IC_50_ with short‐term (72 hours) treatment with ENZ (Figure 1A). As expected, AR‐dependent LNCaP cells were the most sensitive and 22Rv1 cells, which express AR splice variants lacking the ligand‐binding domain, were the most resistant. C4‐2 cells were more sensitive than 22Rv1, however, the IC_50_ significantly exceeds a clinically achievable dose. Analysis of clonogenic survival upon extended treatment (14 days) with ENZ revealed a similar trend as the viability data, where LNCaP cells are most sensitive and 22Rv1 most resistant (Figures 1B,S1). To directly assess the role of apoptosis in response to ENZ, annexin‐V staining was measured by flow cytometry (Figure 1C). Strikingly, the amount of apoptotic cell death in response to ENZ was inversely proportional to viability and survival in the cell lines tested. These results are consistent with the limited responses observed in CRPC treated with AR antagonists and suggest apoptosis inactivation as a potential mechanism of therapeutic resistance.

We recently demonstrated that extrinsic pathway signaling is required for enzalutamide‐induced apoptosis. We showed that death receptor engagement with TNF‐α mediates activation of caspase 8 in response to combination treatment with IAP inhibitor and ENZ, and this signaling is required for apoptotic response.^5^ The mitochondrial apoptosis pathway can be initiated in response to various cellular stressors such as DNA damage and hormone withdrawal.^11^ Importantly, engagement of the extrinsic apoptosis pathway can also activate mitochondrial apoptosis, providing a mechanism to amplify the apoptotic signal through activation of both pathways.^12^ This mechanism is initiated via death receptor binding and activation of caspase 8, leading to BID cleavage and oligomerization of BAX/BAK. We, therefore, examined the activation of mitochondrial apoptosis in response to AR antagonism in prostate cancer cell lines. To assess MOMP in response to ENZ, we measured mitochondrial membrane potential (ΔΨ_m_) via JC‐1staining (Figure 1D). As shown, we observed a reduction of JC‐1 aggregates in LNCaP cells, indicating reduced ΔΨ_m_ in response to ENZ. Consistent with the reduced apoptotic response observed in C4‐2 and 22Rv1, the membrane potential is not significantly reduced in these cell lines. Evaluation of caspase‐9 activation in response to ENZ demonstrates more than two‐fold activation in LNCaP cells, but no significant activation in C4‐2 and 22Rv1 cells (Figure 1E). Furthermore, the level of caspase‐9 activation increases with increasing dose of ENZ in LNCaP cells, while this dose‐dependent effect is minimal in the C4‐2 cells and not observed 22Rv1 cells (Figure S2). Taken together, these findings support the hypothesis that apoptosis is a critical determinant of ENZ sensitivity and activation of the mitochondrial pathway is likely a key mechanism of apoptotic response in prostate tumors.

### 3.2 CRPC cells demonstrate a BCL2 family expression pattern predictive of high apoptotic threshold

**Figure 2:**
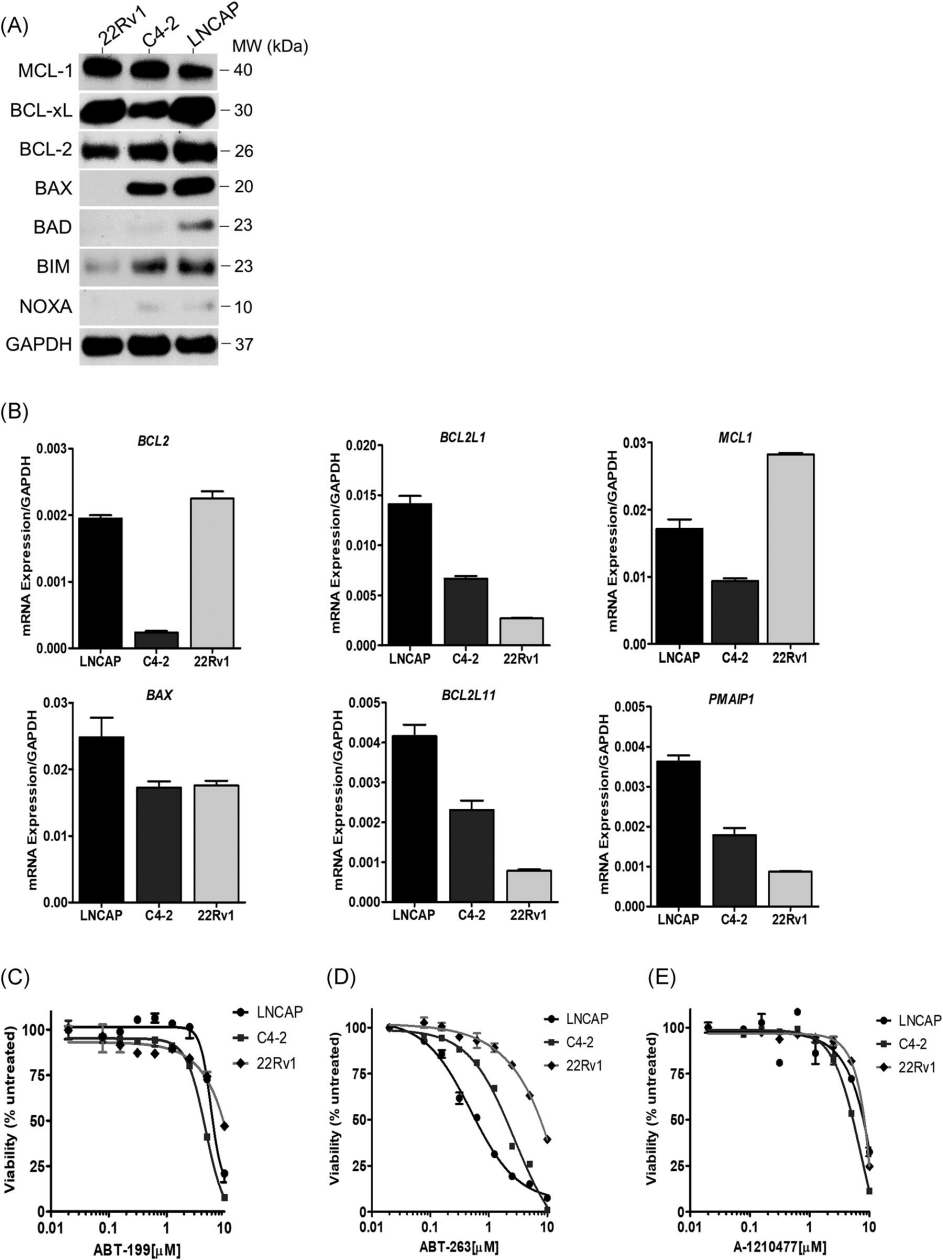
Baseline expression levels of BCL2 family members and sensitivity to BH3 mimetics in prostate cancer cell lines. A, BCL2 family protein expression. GAPDH was used as a loading control. B, BCL2 family gene expression. mRNA transcript levels were normalized to *GAPDH*. C‐E, Viability was measured in cells exposed to increasing concentrations of ABT‐199 (C), ABT‐263 (D), or A‐1210477 (E) for 72 hours and shown as percent of vehicle‐treated control. Values represent the mean ± SEM from three independent experiments. GAPDH, glyceraldehyde 3‐phosphate dehydrogenase; mRNA, messenger RNA

To further investigate mitochondrial apoptosis signaling in the prostate cancer cell line models, we evaluated the expression levels of the BCL2 family signaling effectors that regulate mitochondrial apoptosis activation. Protein expression analysis in the indicated prostate cancer cell lines revealed differences in several proapoptotic and antiapoptotic BCL2 family proteins (Figure 2A). Interestingly, while LNCaP cells show strong expression of antiapoptotic BCL‐2 and BCL‐xL, they also express high levels of proapoptotic BAX and BIM and detectable expression of BAD and NOXA. Conversely, 22Rv1 cells express high levels of BCL‐xL and MCL‐1, while demonstrating undetectable expression of proapoptotic proteins BAX, BIM, BAD, and NOXA. Similarly, C4‐2 cell expresses high levels of BCL‐2, BCL‐xL, and MCL‐1 but reduced expression of BAX and BIM, and undetectable expression of BAD and NOXA. Gene expression analysis of the corresponding BCL2 family genes showed some correlation with the protein expression data, with LNCaP cells demonstrating higher transcript levels of *BAX*, *BCL2L11*, and *PMAIP1* as compared to C4‐2 and 22Rv1 (Figure 2B). These findings are consistent with the mechanism of mitochondrial apoptosis regulation, where the balance of proapoptotic and antiapoptotic BCL2 proteins determine the apoptotic threshold. Applying this concept to the expression pattern observed here suggests that LNCaP sensitivity to ENZ may be related to a lower apoptotic threshold.

To identify whether prostate cancer cells depend on antiapoptotic BCL2 family proteins for cell survival, we tested the activity of three different BH3 mimetics in LNCaP, C4‐2, and 22Rv1 cells. The BH3 mimetics tested include BCL2‐specific inhibitor venetoclax (ABT‐199), the broad‐spectrum inhibitor navitoclax (ABT‐263) which demonstrates activity against BCL‐2, BCL‐xL, and BCL‐w, and the MCL‐1 inhibitor A‐1210477. Viability was measured in each cell line treated with the indicated BH3 mimetic. Single‐agent ABT‐199 demonstrated minimal antiproliferative activity in all the cell lines tested indicating that BCL‐2 is not a critical survival protein in the prostate cancer cells tested (Figure 2C). Treatment with single‐agent ABT‐263 revealed LNCaP cells was exquisitely sensitive to BCL‐xL inhibition, while the antiproliferative response in C4‐2 cells required a higher dose. 22Rv1 cells were insensitive to ABT‐263 single‐agent exposure (Figure 2D). Finally, treatment with MCL‐1 inhibitor A‐1210477 did not affect viability in any of the cell lines tested (Figure 2E). Clonogenic survival in response to the BH3 mimetics was concordant with the viability data (Figure S3‐S5). Taken together, LNCaP and C4‐2 cells demonstrated the greatest responses to single‐agent ABT‐263, but not ABT‐199, suggesting these cell lines are dependent on BCL‐xL for survival. Furthermore, our data reveal that baseline expression of BCL‐2, BCL‐xL or MCL‐1 were not predictive of sensitivity to the BH3‐mimetics tested, similar to studies in hematological malignancies and solid tumors.^13^, ^14^

### 3.3 BH3 mimetics activate apoptosis in CRPC cells treated with AR antagonist

Limited responses to single‐agent BH3 mimetics is frequently observed in solid tumors suggesting protumorigenic survival signaling is not overcome with BH3 mimetic treatment alone. Interestingly, the concept of “priming” cells for apoptosis has been demonstrated in breast cancer cell lines where exposure to chemotherapy or estrogen receptor antagonist resulted in increased BCL‐2 activity and sensitized cells to ABT‐199.^15^, ^16^ To explore the impact of ENZ on BCL2 family expression, we evaluated protein and messenger RNA (mRNA) expression in response to ENZ. Analysis of protein expression, shown in Figure 3A, demonstrates a slight increase in MCL‐1 protein expression in 22Rv1 cells. C4‐2 cells show decreased BCL‐xL in response to ENZ, however minimal changes are observed in the other proteins analyzed in this cell line. Strikingly, in LNCaP cells, BCL‐2 expression is significantly increased in response to ENZ.

Figure 3BH3‐mimetics targeting specific BCL2 proteins enhances the activity of enzalutamide. A, BCL2 family protein expression analysis in cells treated with vehicle or 20 µM ENZ. Tubulin was used as a loading control. B‐D, BCL2 family gene expression in 22Rv1 (B), C4‐2 (C), and LNCaP (D) cells exposed to 20 µM ENZ mRNA transcript levels were normalized to *GAPDH*. E, G, I, Viability of LNCaP (E), C4‐2 (G), or 22Rv1 (I) cells treated for 72 hours with 20 µM ENZ plus 1 µM ABT‐199, 1 µM ABT‐263, or 1 µM A‐1210477. F, H, J, Apoptosis determined by cleaved PARP‐1 expression in LNCaP (F), C4‐2 (H), or 22Rv1 (J) treated with vehicle, 20 µM ENZ, 1 µM of the indicated BH3 mimetic, or combination. GAPDH was used as a loading control. Values represent the mean ± SEM from three independent experiments. ENZ, enzalutamide; GAPDH, glyceraldehyde 3‐phosphate dehydrogenase; mRNA, messenger RNA. *P < .05

**Figure:**
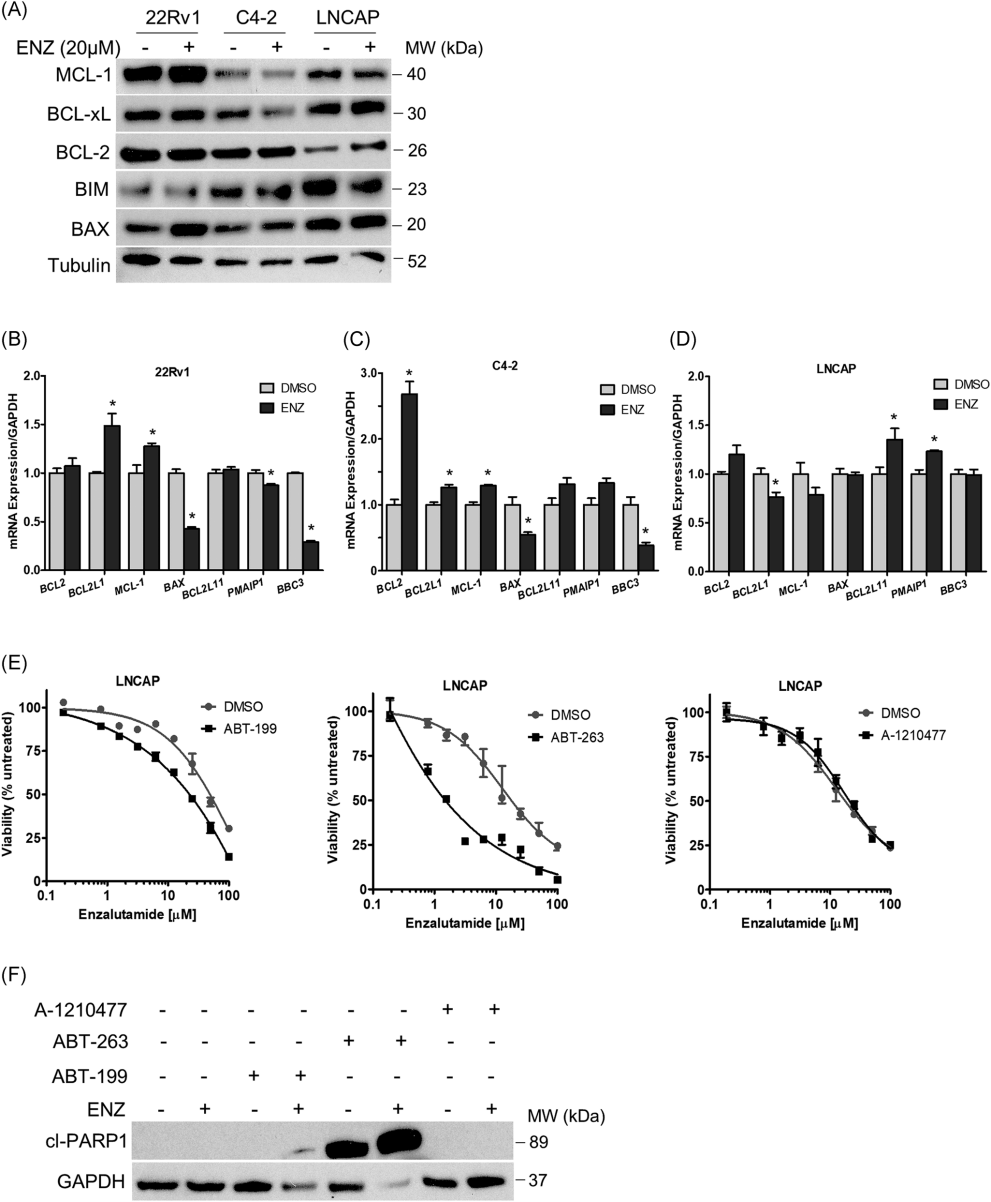

**Figure:**
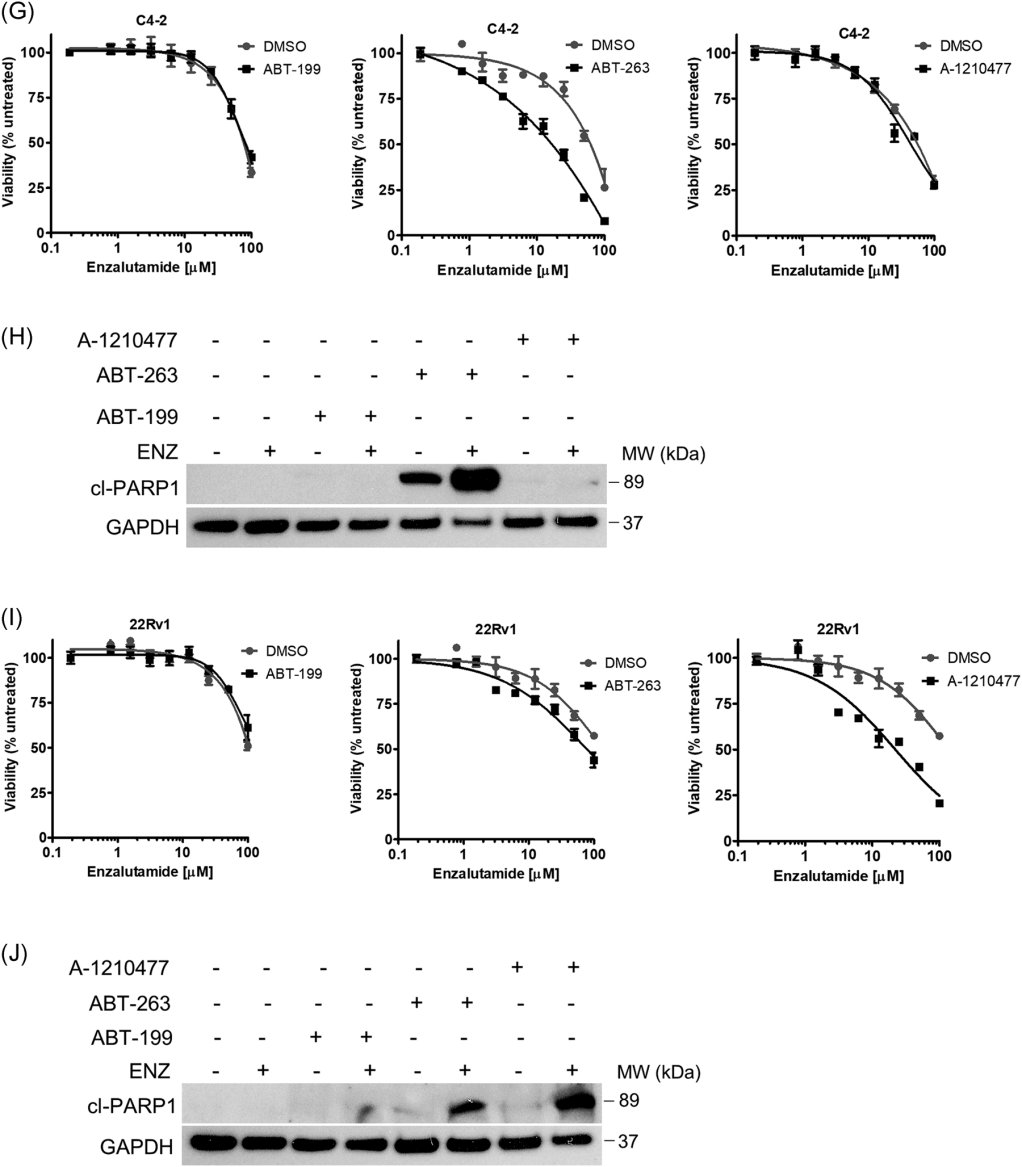

Gene expression analysis demonstrated significant changes in response to ENZ (Figure 3B‐D). Here, ENZ treatment in C4‐2 and 22Rv1 cells induces significant upregulation of antiapoptotic *BCL2*, *BCL2L1*, and *MCL1* and decreased expression of proapoptotic *BAX*, *PMAIP1*, and *BBC3* (encoding proteins NOXA and PUMA, respectively). In contrast, LNCaP cells show decreased expression of antiapoptotic *BCL2L1* and *MCL1* along with increased proapoptotic *BCL2L11* and *PMAIP1* expression upon treatment with ENZ. These results suggest that apoptosis threshold can be modulated in response to ENZ, where sensitive cells are “primed” for apoptosis, likely contributing to a greater response to ENZ.

We, therefore, hypothesized that sensitivity to ENZ reflects proximity to an apoptotic threshold that is determined by BCL2 protein signaling. To identify the BCL2 proteins that promote survival in response to ENZ, we systematically treated cells with the BH3‐mimetics tested above (designed to target BCL‐2, BCL‐xL, or MCL‐1) in combination with ENZ and measured viability and apoptosis. Analysis of LNCaP cells in Figure 3E demonstrates reduced viability in response to the ABT‐199 and ENZ combination, consistent with increased BCL‐2 protein expression observed above, suggesting LNCaP cells exposed to ENZ were primed for apoptosis via BCL‐2 signaling. LNCaP cells were extremely sensitive to the combination of ABT‐263 plus ENZ but did not demonstrate any change in sensitivity to ENZ with the addition of MCL1 inhibitor A‐1210477. To determine the critical BCL2 signaling blocking apoptosis in response to ENZ in LNCaP cells, we measured the level of PARP‐1 cleavage, a functional marker of apoptosis, in LNCaP cells treated with the BH3 mimetics in combination with ENZ (Figure 3F). In line with the viability data, we observe significant PARP‐1 cleavage in response to ABT‐263 alone, and in combination with ENZ. Furthermore, combined treatment with ABT‐199 and ENZ showed a faint cleavage band, indicating an increased apoptotic response with the addition of ABT‐199 in the LNCaP cells. This data indicates that BCL‐2 and BCL‐xL inhibition further sensitize LNCaP cells to ENZ. Consistent with the viability data, the addition of MCL‐1 inhibitor did not increase apoptosis in response to ENZ in the LNCaP cells.

In C4‐2 cells, the addition of ABT‐263 significantly reduces viability in response to ENZ (Figure 3G). Viability is unchanged with the addition of ABT‐199 or MCL1 inhibitor in this cell line. Analysis of apoptosis by PARP‐1 cleavage as shown in Figure 3H, demonstrates robust cleavage with ABT‐263 and ENZ, in agreement with the viability data and further confirming that BCL‐xL is critical for survival in the C4‐2 cells. Viability analysis in 22Rv1 cells demonstrates a modest increase in sensitivity to ENZ with the addition of ABT‐263. Furthermore, a significant increase in sensitivity to ENZ is observed upon addition of MCL1 inhibitor (Figure 3I). Consistent with the viability data, 22Rv1 cells show detectable PARP‐1 cleavage with combined treatment of ABT‐263 plus ENZ and demonstrate stronger PARP‐1 cleavage expression in cells treated with ENZ plus MCL1 inhibitor (Figure 3J).

Taken together, these results reveal that BCL‐xL and MCL‐1 proteins block apoptosis in the CRPC cells in response to AR antagonism. We demonstrate that these proteins can be targeted with BH3 mimetics and sensitizes the CRPC cells to ENZ. Additionally, targeting antiapoptotic BCL2 signaling further sensitizes LNCaP cells to ENZ, demonstrating a potential strategy to prevent acquired drug resistance in initially sensitive tumor cell populations.

### 3.4 BH3 mimetics disrupt BCL2 protein interactions to activate proapoptosis signaling in CRPC cells treated with AR antagonist

**Figure 4:**
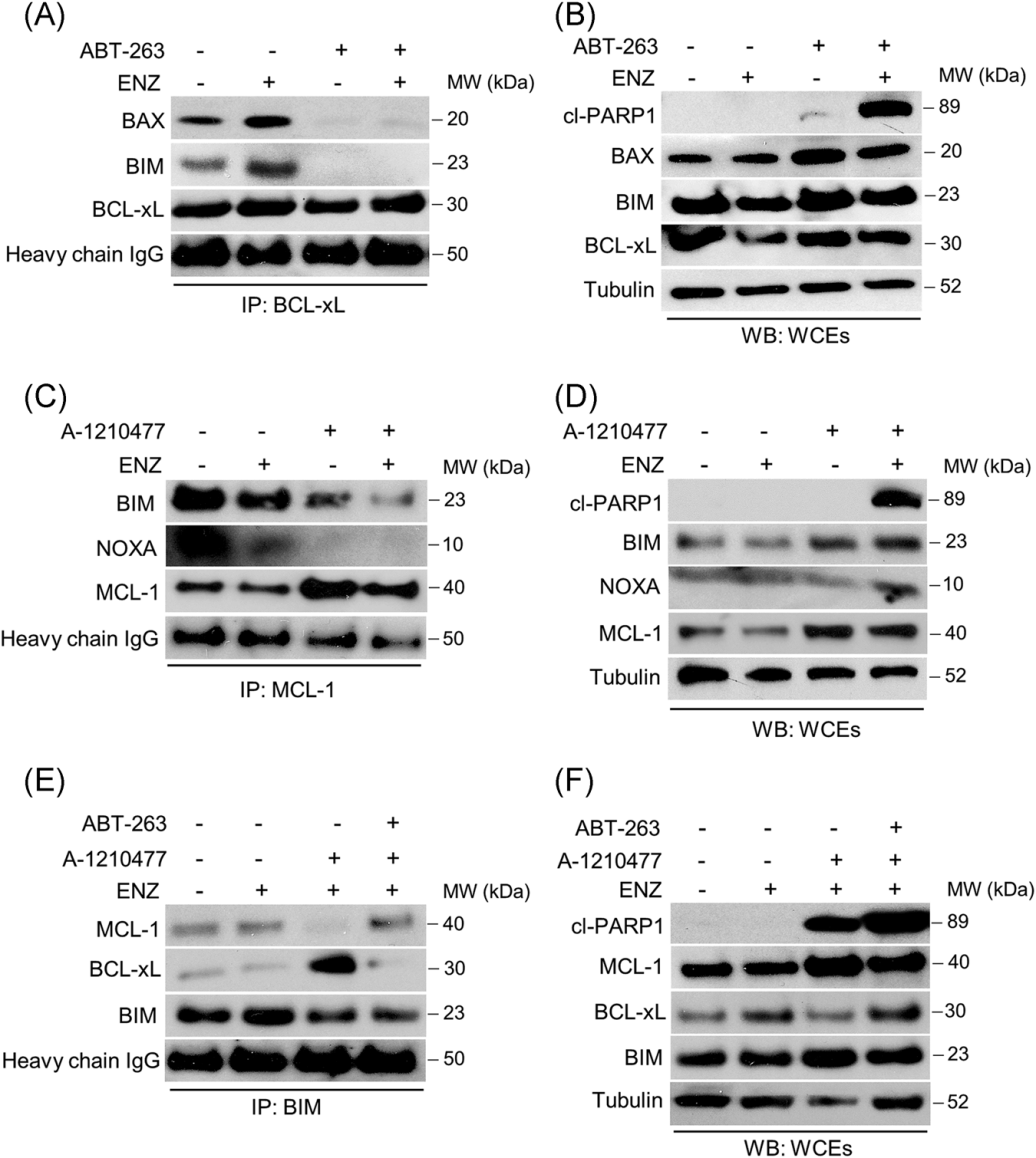
BH3 mimetics disrupt antiapoptotic BCL2 protein interactions and induce cell killing in CRPC cells treated with enzalutamide. A, C, E, Coimmunoprecipitation performed as described in Section 2, analyzed by Western blot with the indicated antibodies. A, Immunoprecipitation (IP) of C4‐2 cells with BCL‐xL antibody following 6 hours treatment with vehicle, 20 µM ENZ, 1 µM of ABT‐263, or the combination as indicated. C, IP of 22Rv1 cells with MCL‐1 antibody following 6 hours treatment with vehicle, 20 µM ENZ, 1 µM of A‐1210477, or the combination as indicated. E, IP of 22Rv1 cells with BIM antibody following treatment with 20 µM ENZ plus 1 µM A‐1210477 and 1 µM ABT‐263 as indicated. B, D, F, Apoptosis determined by cleaved PARP‐1 protein expression in whole‐cell extracts (WCEs) not subjected to IP from C4‐2 (B) or 22Rv1 (D, F) cells treated as in the IP experiments. Tubulin was used as a loading control. CRPC, castration‐resistant prostate cancer; ENZ, enzalutamide; IgG, immunoglobulin G

Protein complexes between the BCL2 family proteins are critical in regulating MOMP and activation of apoptosis.^17^ To determine the BCL2 signaling mechanisms blocking apoptosis in the CRPC cell lines, we used immunoprecipitation to identify the BCL2 protein interactions in response to ENZ and BH3 mimetic treatment. We focused on the BH3‐only protein BIM since it is a direct activator of BAX/BAK and a sensitizer (antagonist) to all the antiapoptotic BCL2 protein members.^18^ As shown in untreated C4‐2 cells, both BIM and BAX are complexed with BCL‐xL (Figure 4A). Intriguingly, this interaction increases in response to ENZ, indicating inducible antiapoptotic signaling upon treatment. Treating the cells with ABT‐263 alone or in combination with ENZ disrupts the BCL‐xL interaction with BIM and BAX, thus liberating these proapoptotic proteins. These results correlate with PARP‐1 cleavage with the combination treatment observed in the whole‐cell extracts (WCEs) and support the finding that BCL‐xL is a key antiapoptotic mediator in this CRPC model (Figure 4B).

In 22Rv1 cells, using coimmunoprecipitation we demonstrate that MCL‐1 is complexed with both BIM and NOXA (Figure 4C). Treatment with MCL1 inhibitor alone liberates NOXA but does not completely disrupt the interaction with BIM. However, the addition of ENZ permits the release of BIM and NOXA and correlates with the activation of apoptosis in response to the combination (Figure 4D). Performing the inverse coimmunoprecipitation analysis using BIM confirmed that BIM is in complex with MCL‐1, and this interaction is lost in response to combined treatment with MCL1 inhibitor and ENZ (Figure 4E). Strikingly, however, MCL‐1 inhibition results in a strong induction of BCL‐xL and BIM interaction, indicating that BIM release from MCL‐1 is rapidly sequestered by BCL‐xL. Treatment with a combination of all three inhibitors (ABT‐263, MCL1 inhibitor, and ENZ) results in release of BIM from BCL‐xL and MCL‐1 and correlates with robust activation of apoptosis, as shown by PARP‐1 cleavage in the WCEs (Figure 4F). These findings agree with the viability data in 22Rv1 cells that demonstrate MCL‐1 and BCL‐xL are critical determinants of survival in this CRPC model. Taken together, we mechanistically determined the BCL2 protein interactions that block apoptosis in response to ENZ and that targeted treatment with BH3 mimetics disrupts these interactions and permits apoptosis activation.

### 3.5 PI3K/Akt blockade indirectly activates proapoptosis signaling and sensitizes CRPC cells to AR antagonist

**Figure 5:**
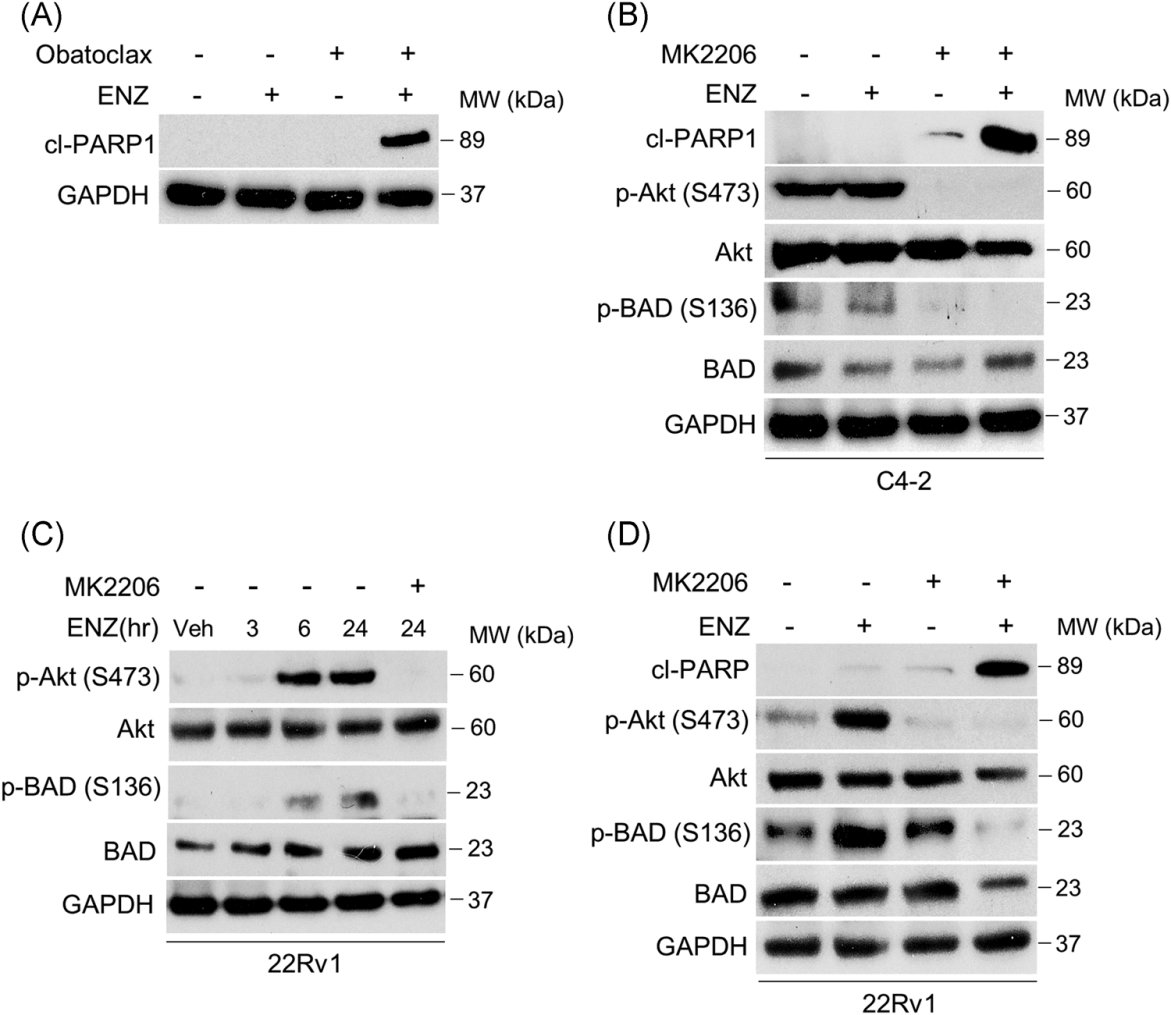
Akt blockade indirectly targets BCL‐xL through BAD activation sensitizing CRPC cells to enzalutamide. A, Apoptosis determined by cleaved PARP‐1 expression in 22Rv1 cells treated with 20 µM ENZ, 0.1 µM obatoclax, or the combination. GAPDH was used as a loading control. B, D, Western blot analysis of C4‐2 (B) or 22Rv1 (D) cells treated with 20 µM ENZ, 1 µM Akt inhibitor MK2206, or the combination. C, 22Rv1 cells subjected to 20 µM ENZ time course treatment and analyzed at 3, 6, and 24 hours with the indicated antibodies. Cells cotreated with 1 µM MK2206 were analyzed at 24 hours. CRPC, castration‐resistant prostate cancer; ENZ, enzalutamide; GAPDH, glyceraldehyde 3‐phosphate dehydrogenase

Our results demonstrate that BCL‐xL and MCL‐1 are critical for antiapoptotic signaling in response to ENZ. Furthermore, we show that BCL‐xL and MCL‐1 can function cooperatively to sequester proapoptotic proteins and prevent apoptosis, demonstrating that dual inhibition of both proteins is required for maximum apoptotic response and increased ENZ sensitivity. Since the translational feasibility of using a triplet treatment approach is currently unlikely, we tested obatoclax, a pan‐BCL2 inhibitor with activity against BCL‐2, BCL‐xL, and MCL‐1 in combination with ENZ (Figure 5A). As shown, combined treatment with obatoclax potently induces apoptosis in 22Rv1 cells. However, due to severe toxicity obatoclax has been eliminated from further clinical development.^19^, ^20^

To identify a potential strategy for inhibition of BCL‐xL, we considered its biological antagonist, BAD. The proapoptotic function of BAD is negatively regulated through phosphorylation by Akt, where upstream activation of Akt leads to inhibitory BAD phosphorylation at Ser136 and subsequent 14‐3‐3 sequestration.^21^ Since PTEN is frequently deleted in advanced prostate cancer, and results in constitutive activation of the PI3K/Akt pathway, we hypothesized that inhibition of BAD by Akt is a potential apoptosis evasion mechanism in prostate tumors. We tested this in PTEN‐deleted C4‐2 cells that demonstrate constitutively active PI3K/Akt signaling. As shown in C4‐2 cells, activated Akt (demonstrated by phosphorylation at Ser473) correlates with inactivating phosphorylation of BAD at Ser136 (Figure 5B). Treatment with Akt inhibitor MK2206 abolishes Akt activity and prevents downstream BAD phosphorylation, resulting in apoptosis in combination with ENZ. We have shown that 22Rv1 cells display dependency on BCL‐xL for survival upon exposure to ENZ, albeit less than PTEN mutated cells C4‐2 and LNCaP. To evaluate whether BAD inactivation played a role in apoptosis resistance in PTEN–wild‐type 22Rv1 cells, we assessed PI3K/Akt signaling in response to ENZ. As expected, PI3K/Akt is not active in 22Rv1 cells at baseline, likely due to intact PTEN blocking constitutive PI3K signaling. Strikingly, Akt is activated in the 22Rv1 cells upon treatment with ENZ, and this activation is PI3K‐dependent (Figure S6). We, therefore, asked whether induced Akt activation in response to ENZ could inactivate BAD, resulting in a BCLxL‐mediated apoptosis evasion mechanism in PTEN‐wild‐type prostate tumor cells. Indeed, in response to ENZ we observe activation of Akt displaying similar kinetics to inactivating Ser136 phosphorylation of BAD (Figure 5C). Consistent with our observations in C4‐2 cells, inhibition of Akt kinase activity with MK2206 prevents downstream BAD phosphorylation and induces apoptosis in combination with ENZ (Figure 5D). Together, these results demonstrate that PI3K/Akt signaling inactivates proapoptotic signaling in CRPC cells and targeting this alternative pathway is a potential strategy to increase apoptotic response in both PTEN‐mutant and PTEN–wild‐type prostate tumors.

## 4 DISCUSSION

**Figure 6:**
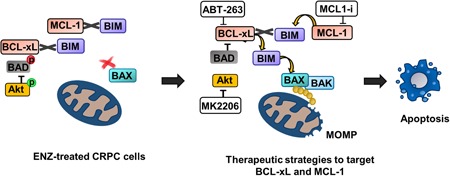
Schematic displaying the antiapoptotic protein interactions in ENZ‐treated CRPC cells. Strategies to disrupt these interactions by targeting BCL‐xL and MCL‐1 with ABT‐263 and MCL1 inhibitor (MCL1‐i), respectively is depicted on the right. The approach to inhibit BCL‐xL signaling through BAD activation via Akt blockade is also shown. CRPC, castration‐resistant prostate cancer; ENZ, enzalutamide; MOMP, mitochondrial outer membrane permeabilization

CRPC is a heterogeneous disease with multiple mechanisms contributing to treatment resistance and disease progression. AR‐targeted therapy is critical for the treatment of CRPC, however, responses are limited by intrinsic and acquired resistance. Although several AR‐related resistance mechanisms have been identified, alternative pathways of resistance remain largely undefined. In this study, we demonstrate that apoptosis evasion through antiapoptotic BCL2 signaling is a mechanism of enzalutamide resistance in CRPC cells. Our findings show that critical BCL2 proteins can be targeted to induce apoptosis and sensitize CRPC cells to enzalutamide. In the CRPC models tested, we determined that BCL‐xL and MCL‐1 are critical antiapoptotic effectors that mediate survival upon exposure to ENZ. Exploring the mechanism of antiapoptotic protein signaling revealed that BCL‐xL and MCL‐1 can be found complexed with BIM and BAX, blocking their proapoptotic function and supporting survival upon treatment with ENZ. Inhibiting BCL‐xL with antagonist ABT‐263 eliminated these protein interactions and liberated BIM and BAX to induce apoptosis. Interestingly, the BIM:MCL‐1 complex could be disrupted through targeting MCL‐1, but resulted in a strong BIM:BCL‐xL complex, indicating a dependence on both MCL‐1 and BCL‐xL for apoptosis evasion and cell survival. A model depicting the impact of ENZ and BH3 mimetic treatment on BCL2 protein signaling is summarized in Figure 6.

The robust apoptotic responses demonstrated by targeting BCL2 proteins in combination with ENZ in the preclinical setting suggests the potential to develop this strategy clinically. We’ve shown here that BCL‐xL is a key prosurvival player in response to ENZ in CRPC cells and is a potential resistance mechanism to MCL1 inhibtion. Therefore, targeting BCL‐xL is likely critical in achieving maximum apoptotic response in combination treatment strategies. To address the dose‐limiting adverse toxicities observed with BCL‐xL‐targeted BH3 mimetics, we identified an alternative strategy to inhibit BCL‐xL through activation of BAD, the endogenous antagonist of BCL‐xL. Akt is found to be constitutively active in PTEN‐deleted tumors, including prostate, and can regulate proapoptotic BAD signaling. In our PTEN‐deleted CRPC cell line, we inhibited Akt kinase signaling, resulting in BAD activation and robust apoptosis in response to ENZ. Interestingly, in PTEN–wild‐type 22Rv1 cells, we discovered PI3K‐dependent activation of Akt upon exposure to ENZ, suggesting activation of PI3K signaling upon AR inhibition may play a role in ENZ resistance. Furthermore, 22Rv1 cells treated with ENZ demonstrate Akt activation with congruent BAD phosphorylation (and inactivation) indicating apoptosis evasion signaling is activated in response to AR inhibition. Similar to the PTEN‐mutant cells, inhibition of Akt allows BAD activation and apoptosis. This proposed approach is summarized in Figure 6. Collectively, the mechanism of BAD inactivation by dysregulated PI3K/Akt signaling may be an important resistance mechanism in both PTEN‐deleted and PTEN–wild‐type prostate cancer and highlights a novel therapeutic strategy to increase apoptotic response to AR antagonists.

There is a critical need to identify superior therapeutic strategies to improve outcomes in men with CRPC. The AR remains an important driver in advanced prostate cancer, however, responses to AR‐targeting therapies in CRPC is limited, with most patients experiencing limited therapeutic responses and rapid disease progression. Moreover, two recent studies examined the antitumor activity of intense androgen deprivation strategies through neoadjuvant ADT plus abiraterone or ADT plus ENZ, with the goal of maximum AR inhibition to translate into greater tumor response.^22^, ^23^ Indeed, intratumoral androgens and AR transcriptional activity were nearly undetectable with treatment, however complete responses were limited, with some patients demonstrating disease progression. These studies indicate that even with a near total reduction of AR activity, the tumor cell population is not eliminated. This suggests that surviving clones persist, likely harboring heterogeneous, bypass survival mechanisms capable of driving drug resistance and disease progression. This highlights that in addition to targeting AR, it is critical to target the alternate survival pathways that drive treatment failure in CRPC.

In this study, we investigated the role of antiapoptotic BCL2 family signaling in resistance to AR antagonist treatment. Several studies in prostate tumors have shown that elevated expression of BCL‐2, BCL‐xL, and MCL‐1 is associated with tumor aggressiveness, treatment resistance, and metastatic progression.^9^, ^24^ Therefore, targeting the antiapoptotic signaling effectors is an attractive strategy to increase apoptotic response in tumors. BH3 mimetics are small molecule inhibitors designed to bind and inhibit antiapoptotic BCL2 proteins to trigger apoptosis.^25^ Several BH3 mimetics have been developed with many currently in clinical trials. To date, the only FDA approved BH3 mimetic is venetoclax (ABT‐199), a platelet‐sparing selective BCL‐2 inhibitor approved for relapsed CLL.^26^, ^27^ MCL‐1 is emerging as a critical prosurvival protein in cancer, with several promising MCL‐1 inhibitors currently in development.^28^, ^29^

Consistent with previous studies, we did not observe BCL‐2, BCL‐xL, or MCL‐1 expression levels to be predictive of response to the BH3 mimetics tested. As shown here, the interactions between proapoptotic and antiapoptotic proteins established the apoptotic threshold, suggesting that baseline expression of antiapoptotic proteins may not provide a robust predictive biomarker. However, higher expression levels of proapoptotic players such as BIM and BAX may offset sequestration and provide a mechanism to detect tumors with a lower apoptotic threshold and predictive of better response. Additionally, we observed sensitivity to BCL‐xL inhibition with single‐agent ABT‐263 in two PTEN‐deleted cell lines, suggesting tumors with activated PI3K/Akt are dependent on BCL‐xL for survival. A recent clinical trial investigating the Akt inhibitor ipatasertib in combination with abiraterone acetate in men with metastatic CRPC showed improved progression‐free survival in patients with PTEN inactivating mutations, supporting the rationale to target Akt.^30^

Intriguingly, we demonstrate that the addition of MCL1 inhibitor significantly increases ENZ sensitivity in 22Rv1 cells, which express the constitutively active AR‐v7 splice variant. Furthermore, protein and mRNA expression of MCL‐1 is increased upon ENZ treatment in these cells, suggesting regulation is at the transcriptional level. MCL‐1 expression is regulated by several mechanisms including PI3K/Akt/CREB activation^31^ and by AR‐dependent activation of Src kinase and its downstream effector STAT3, a transcription factor targeting the MCL‐1 promoter.^32^ AR‐v7 lacks the ligand‐binding domain and is not inhibited by ENZ, suggesting that perpetual AR‐v7 signaling facilitates survival through increased MCL‐1 expression. This highlights the need for further investigation to determine whether MCL‐1 is a critical target in AR‐v7 expressing prostate tumors.

Taken together, the strategy of amplifying apoptosis as a therapeutic approach in CRPC has the potential to maximize tumor cell death, that could translate into longer therapeutic responses, slower disease progression and the possibility of achieving complete responses. Overall, this study demonstrates that targeting antiapoptotic BCL2 protein signaling in combination with AR inhibition results in enhanced tumor cell killing, suggesting this is a promising therapeutic strategy with the potential for clinical development.

## CONFLICT OF INTERESTS

The authors declare that there are no conflict of interests.

## Supporting information

Supporting information

Supporting information

Supporting information

Supporting information

Supporting information

Supporting information
