# Supplementary figures and images for: Targeting prosurvival BCL2 signaling through Akt blockade sensitizes castration‐resistant prostate cancer cells to enzalutamide
Source: Prostate. 2019 Jun 22;79(11):1347–59. doi: 10.1002/pros.23843 (PMC6617752; doi:10.1002/pros.23843)

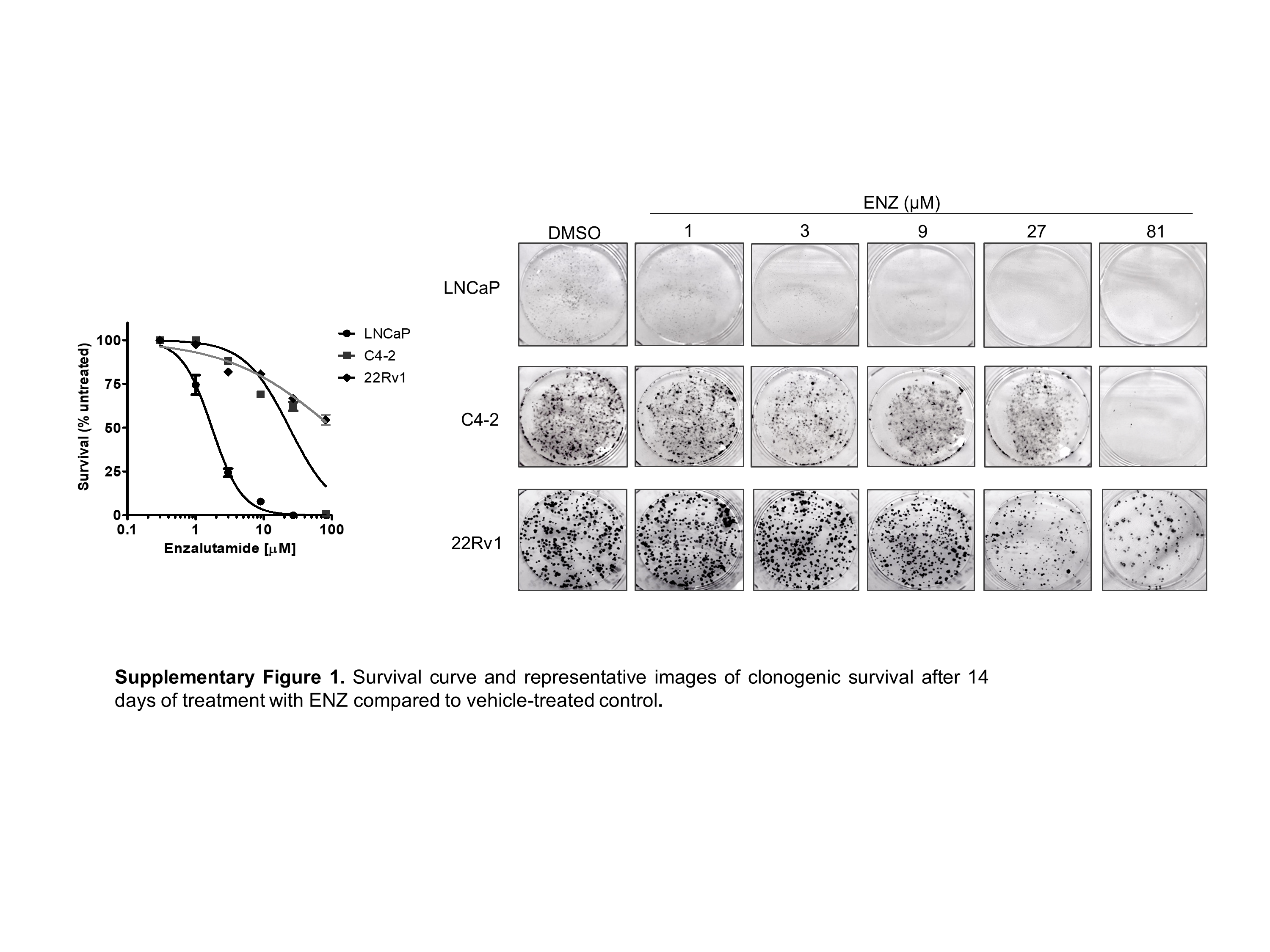

Supplement: Supplementary file 1 — Supporting information [file PROS-79-1347-s001.TIF]

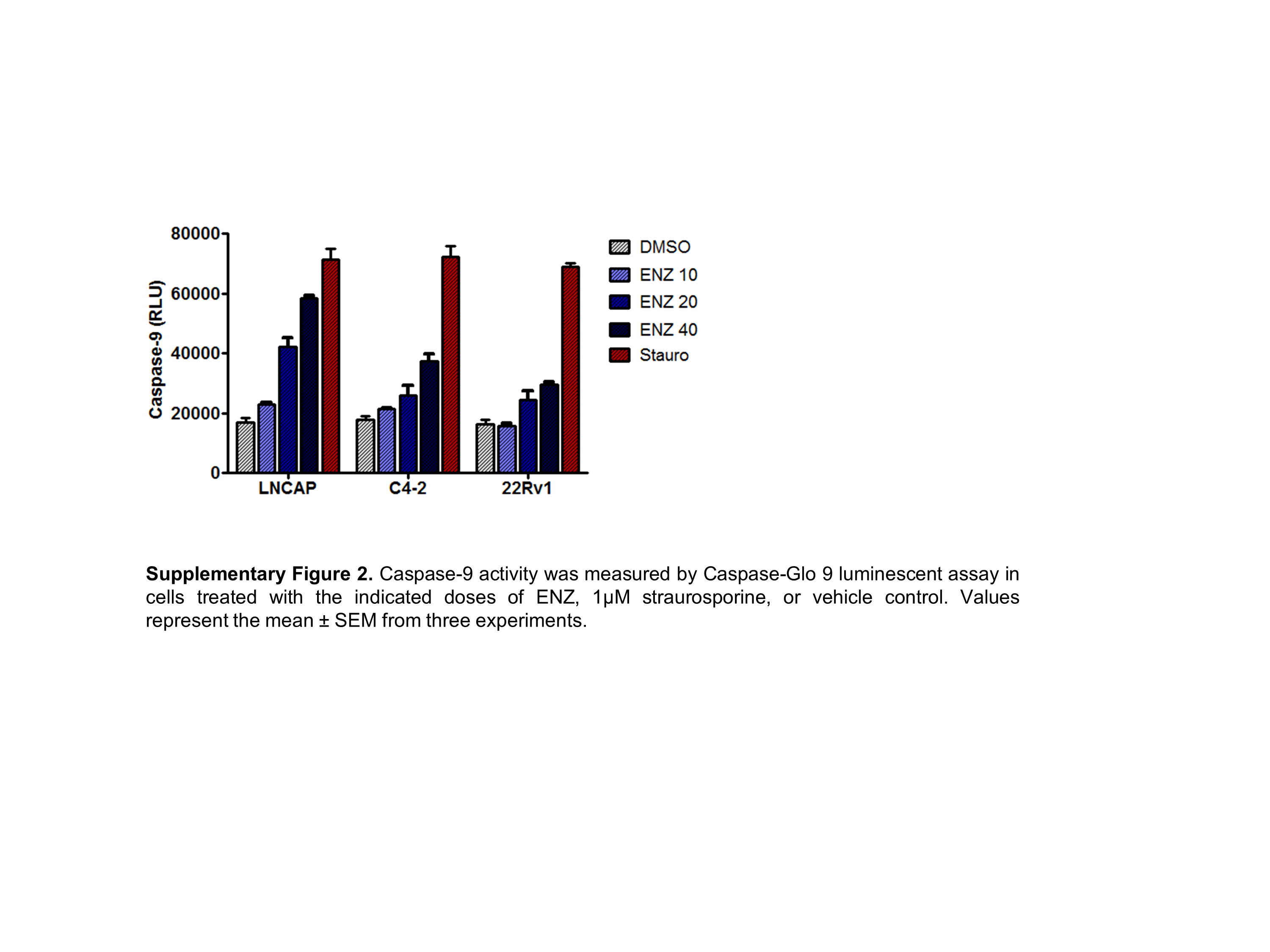

Supplement: Supplementary file 2 — Supporting information [file PROS-79-1347-s002.TIF]

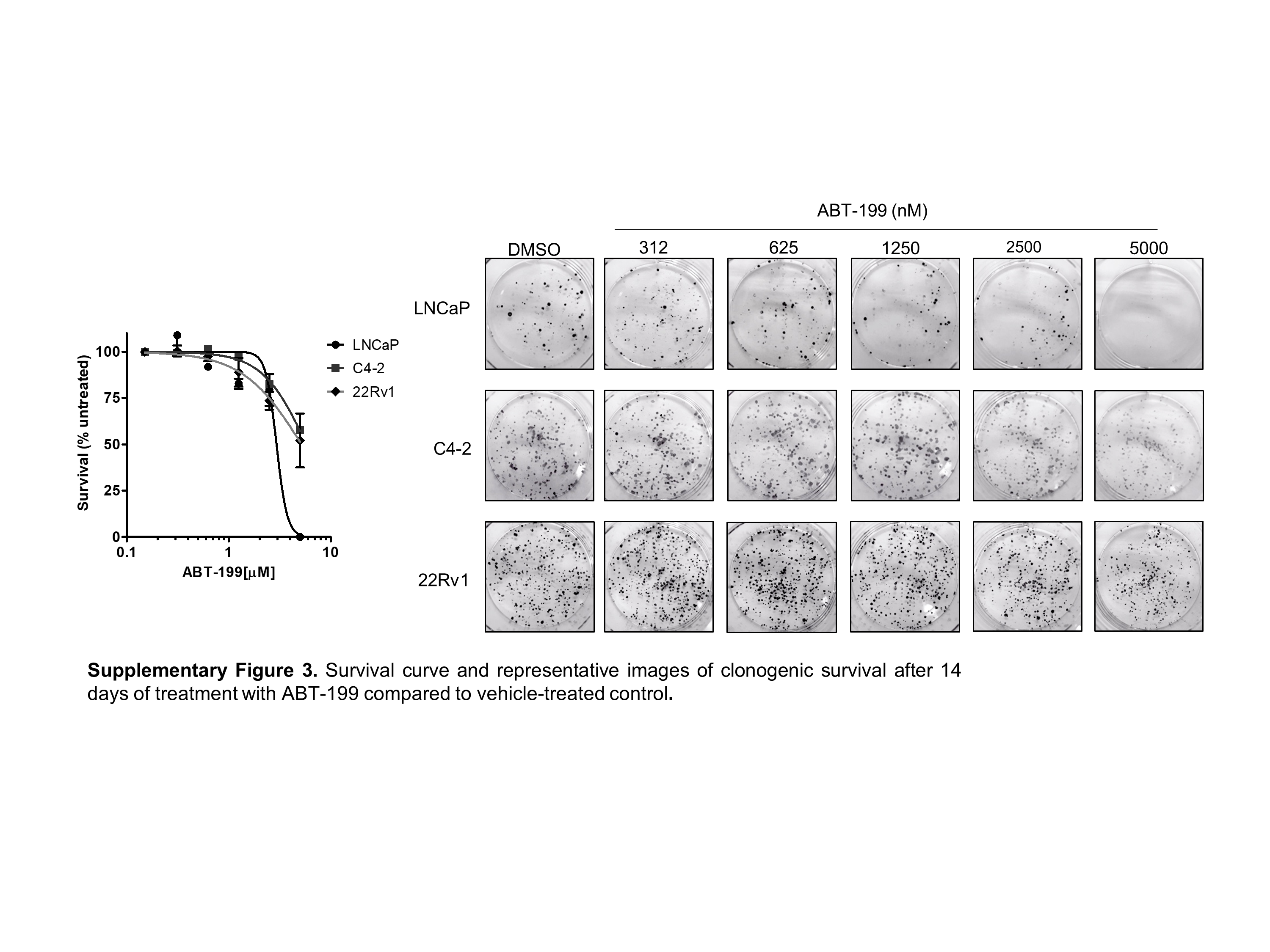

Supplement: Supplementary file 3 — Supporting information [file PROS-79-1347-s003.TIF]

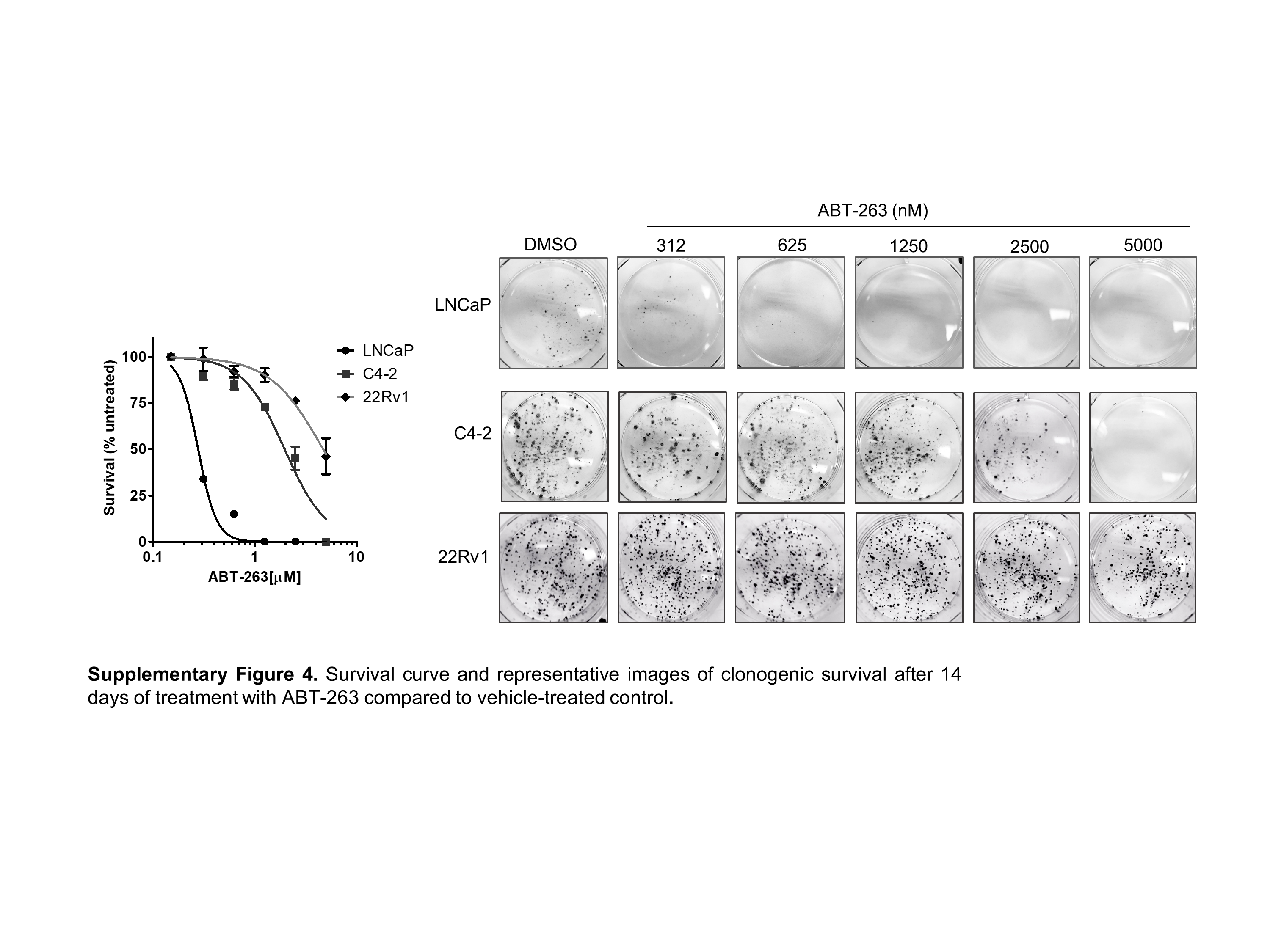

Supplement: Supplementary file 4 — Supporting information [file PROS-79-1347-s004.TIF]

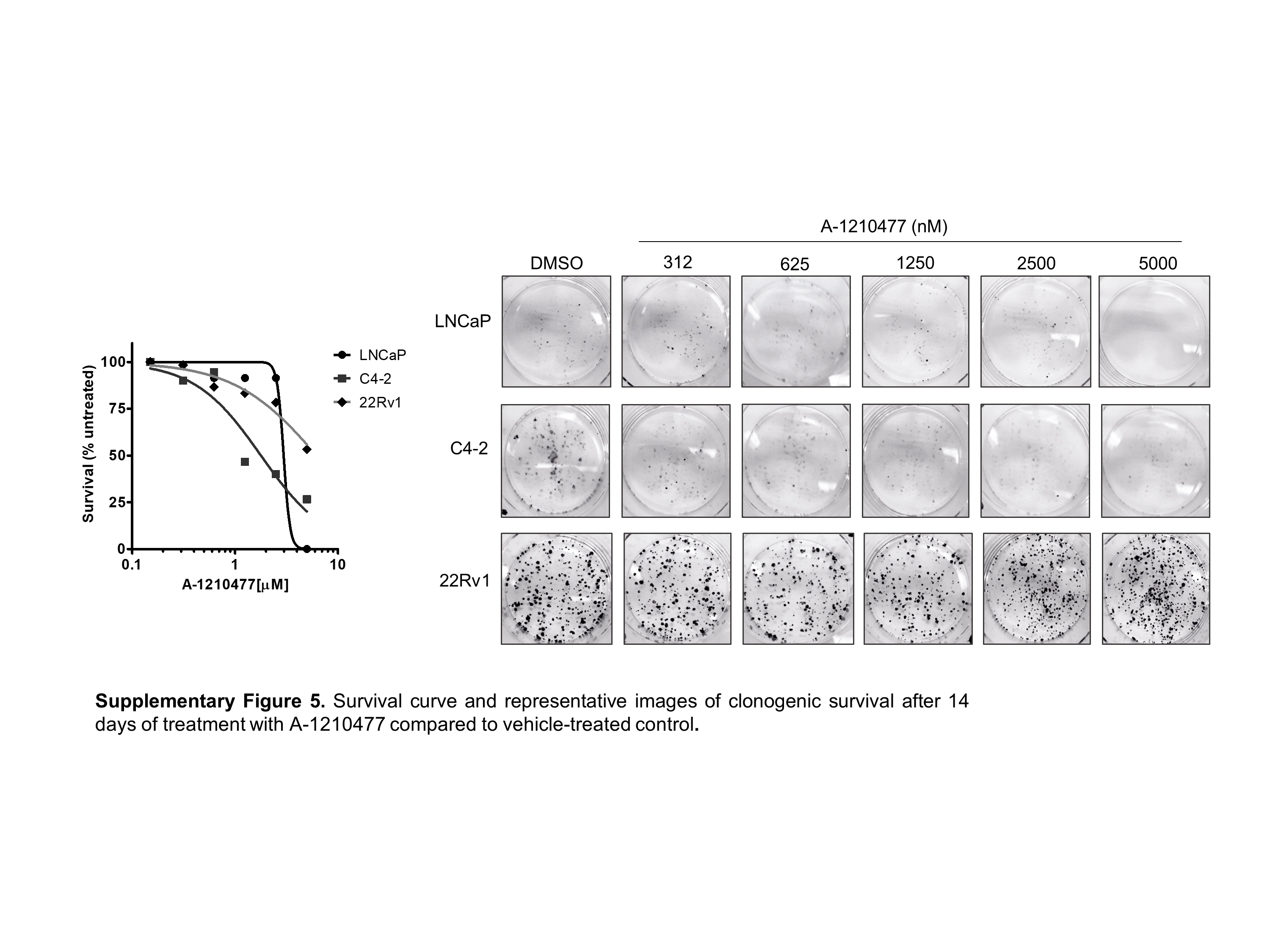

Supplement: Supplementary file 5 — Supporting information [file PROS-79-1347-s005.TIF]

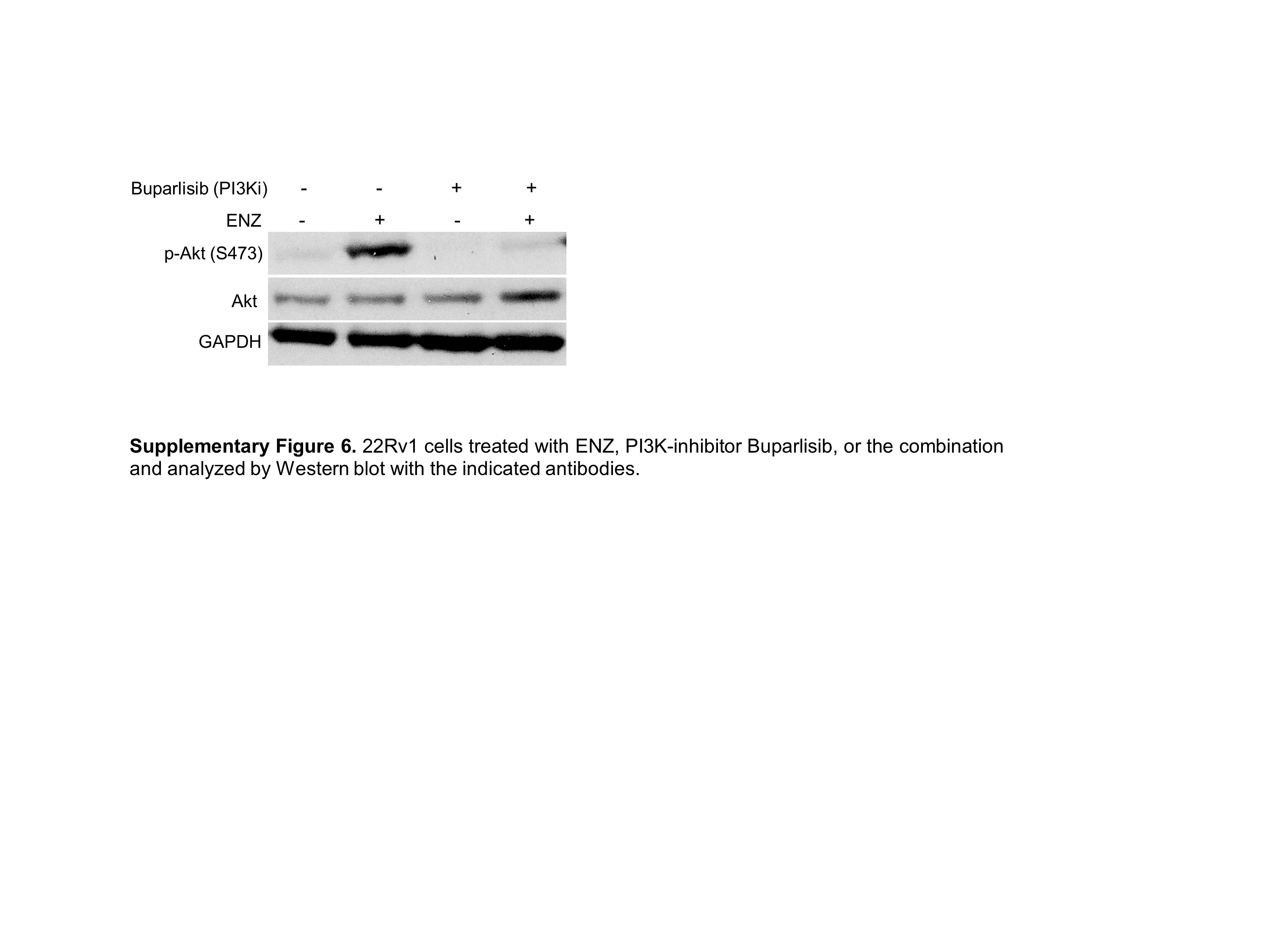

Supplement: Supplementary file 6 — Supporting information [file PROS-79-1347-s006.TIF]
